# Different neighborhood walkability indexes for active commuting to school are necessary for urban and rural children and adolescents

**DOI:** 10.1186/s12966-020-01028-0

**Published:** 2020-09-29

**Authors:** Javier Molina-García, Sergio Campos, Xavier García-Massó, Manuel Herrador-Colmenero, Patricia Gálvez-Fernández, Daniel Molina-Soberanes, Ana Queralt, Palma Chillón

**Affiliations:** 1 Department of Teaching of Musical, Visual and Corporal Expression, University of Valencia, Avda. dels Tarongers, 4, 46022 Valencia, Spain; 2 AFIPS research group, University of Valencia, Valencia, Spain; 3 Department of Urban and Spatial Planning, University of Granada, C/ Severo Ochoa, s/n, 18071 Granada, Spain; 4 “La Inmaculada” Teacher Training Centre, University of Granada, C/ Joaquina Eguaras, 114, 18013 Granada, Spain; 5 Department of Physical Education and Sports, Faculty of Sport Sciences, University of Granada, PROFITH “Promoting FITness and Health through physical activity” research group, Ctra. Alfacar, s/n, 18011 Granada, Spain; 6 Department of Preventive Medicine and Public Health, Faculty of Medicine, University of Granada, Avda. de la Investigación, 11, 18016 Granada, Spain; 7 Department of Nursing, University of Valencia, Jaume Roig, s/n, 46010 Valencia, Spain

**Keywords:** School active travel, Active transport, Youth, Physical environment, MAPS-global tool, Physical activity, Health disparities

## Abstract

**Background:**

Literature focusing on youth has reported limited evidence and non-conclusive associations between neighborhood walkability measures and active commuting to and from school (ACS). Moreover, there is a lack of studies evaluating both macro- and micro-scale environmental factors of the neighborhood when ACS is analyzed. Likewise, most studies on built environment attributes and ACS focus on urban areas, whereas there is a lack of studies analyzing rural residential locations. Moreover, the relationship between built environment attributes and ACS may differ in children and adolescents. Hence, this study aimed to develop walkability indexes in relation to ACS for urban and rural children and adolescents, including both macro- and micro-scale school-neighborhood factors.

**Methods:**

A cross-sectional study of 4593 participants from Spain with a mean age of 12.2 (SD 3.6) years was carried out. Macro-scale environmental factors were evaluated using geographic information system data, and micro-scale factors were measured using observational procedures. Socio-demographic characteristics and ACS were assessed with a questionnaire. Several linear regression models were conducted, including all the possible combinations of six or less built environment factors in order to find the best walkability index.

**Results:**

Analyses showed that intersection density, number of four-way intersections, and residential density were positively related to ACS in urban participants, but negatively in rural participants. In rural children, positive streetscape characteristics, number of regulated crossings, traffic calming features, traffic lanes, and parking street buffers were also negatively related to ACS. In urban participants, other different factors were positively related to ACS: number of regulated crossings, positive streetscape characteristics, or crossing quality. Land use mix acted as a positive predictor only in urban adolescents. Distance to the school was a negative predictor on all the walkability indexes. However, aesthetic and social characteristics were not included in any of the indexes.

**Conclusions:**

Interventions focusing on improving built environments to increase ACS behavior need to have a better understanding of the walkability components that are specifically relevant to urban or rural samples.

## Background

Current evidence indicates that many children and adolescents worldwide are insufficiently physically active, with only 19% meeting physical activity recommendations of daily 60-min moderate-to-vigorous physical activity [1]. Active commuting to and from school (ACS; i.e., walking or cycling) represents an opportunity to increase overall physical activity among children and adolescents [2, 3]. It is related to several health benefits, such as better health-related fitness and psychological well-being [4, 5]. Nevertheless, several studies have shown that there is a decrease in ACS behavior in young people, mainly during adolescence [6–8].

From an ecological perspective [9], personal, psychosocial, and environmental factors and policies influence ACS behavior. Recently, the role of physical environmental factors in ACS has been examined with great interest [10, 11]. The concept of walkability was developed to explain how friendly an area is to active commuting behaviors [12]. Researchers focusing on children and adolescents have reported limited evidence and non-conclusive associations between neighborhood walkability measures and ACS [11, 13]. Apart from the small number of studies on this topic [11], the inconsistent associations across studies might be due to the differences in the methods used to assess built environment attributes [13, 14]. The associations between built environments and physical activity are more consistent when studies are based on objective neighborhood evaluation methods, such as geographical information system (GIS) or observational tools, compared to subjective methods (perception instruments) [14]. Likewise, there is a wide variety of variables to be considered when walkability is evaluated in relation to ACS [10]. The most usual components considered to calculate walkability are street connectivity, residential density, and land use mix [15–17]. A walkability index is calculated by combining these components into a composite value [17]. These neighborhood environment components are considered macro-scale features because they are structural and urban form attributes that are not easily modifiable [18]. More connected areas are hypothesized to promote ACS because there are more route choices through the street network [19]. In this regard, the number of four-way intersections would also be related to the ease of ACS [20]. Moreover, higher residential density has also been related to more active commuting behaviors because it provides more opportunities for social interactions, more commercial activity, or better public transportation systems [21, 22]. Literature has not found consistent results for positive or negative associations between land use diversity and ACS across countries from different continents in children or adolescents [11, 13].

In addition, micro-scale features are smaller details of built environments that can be modified more easily [18, 23], such as street-crossing quality, the presence of parking areas, traffic calming features, and aesthetic and social characteristics. Micro-scale factors are believed to affect people’s experience of being active in a neighborhood in terms of confidence or comfort [23, 24]. The very limited literature on this topic has shown that micro-scale attributes are strongly associated with active commuting behaviors across the lifespan [24]. However, no clear associations have been found with these types of environmental factors when ACS has been specifically analyzed in young people [25].

Currently, there is a lack of studies evaluating both macro- and micro-scale factors of the neighborhood when analyzing ACS [25, 26]. More specifically, the use of composite measures (i.e., walkability indexes) that include these two types of factors is virtually non-existent. In this regard, because macro- and micro-scale attributes can influence ACS behaviors, it would be interesting to have walkability indexes that combine these two types of factors. The creation of these indexes could provide more detailed information about the role of the built environment in ACS behavior. Furthermore, most studies on built environment attributes and ACS focus on urban areas [10], whereas there is a lack of studies analyzing rural residential locations [11]. Hence, it is necessary to know which built environment factors are the greatest determinants in each type of environment. Moreover, the relationship between built environment attributes and ACS may differ in children or adolescents [11, 17, 27].

A comprehensive understanding of the built environment attributes is necessary to inform evidence-based interventions to increase ACS in children and adolescents from different residential areas. Consequently, the aim of this study was to examine the association between the objectively measured school-neighborhood environment and ACS in samples of urban and rural children and adolescents. In addition, several walkability indexes combining both macro- and micro-scale environmental factors were developed and validated for each sample.

## Methods

### Study design, participants, and procedure

This cross-sectional study was carried out in 2012. The initial sample was composed of 6979 children (aged 7–18 years old) from 39 schools from Southeastern regions of Spain (i.e., Almería, Granada, and Murcia). Both primary-school children and secondary-school adolescents were recruited as a convenience sample. The participants who had not completed data on their mode of transport to or from school and/or their family postal address were excluded from the study (*n* = 975). The sample was categorized into two groups according to the participant’s type of residential area (i.e., urban or rural). Municipalities were classified as urban areas if they had ≥20,000 residents, and rural areas if they had < 20,000 residents [28, 29].

**Fig. 1:**
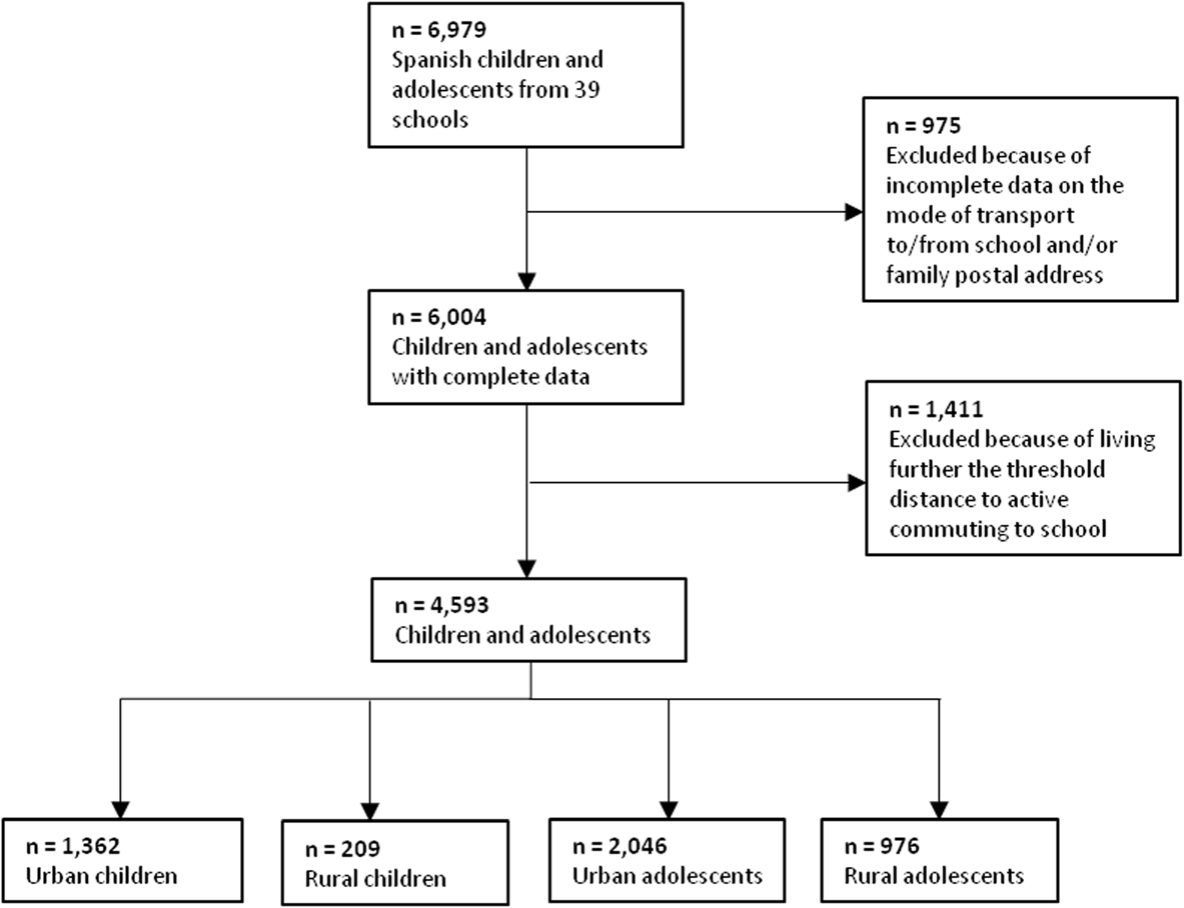
Flowchart of study participants

Participants who lived farther than the threshold distance for ACS according to previous research [30] were excluded (*n* = 1411). The threshold distance is the distance from which the number of passive commuters to school exceeds the number of active commuters [31]. Therefore, 4593 participants (50.7% male) were included in the final sample, with a mean age of 12.2 years (SD 3.6) (Fig. 1). The distribution of the sample by age and residential area was: urban children (*n* = 1362), rural children (*n* = 209), urban adolescents (*n* = 2046), and rural adolescents (*n* = 976).

### Measures

#### Active commuting to and from school

To assess the commuting behavior to and from school, a valid questionnaire was used [32]. The questionnaire was self-completed, requiring about 15 min. This instrument includes questions about how they had commuted to and from school every day during the previous week (i.e., “How did you get to school each day?” and “How did you get home from school each day?”), as well as questions about personal data (i.e., date of birth, gender, postal address, school, and grade). The response options for both questions about ACS were walking; cycling; by motorcycle, car, or bus; and other. A variable was created from the sum of each active trip (i.e. walk and cycle), ranging from 0 to 10 trips per week.

#### Macro-scale variables of the school neighborhood environment

Characteristics of the neighborhood environments around schools were calculated using GIS. Initially, the shortest distance along the street network between home and school was calculated. School neighborhoods were defined by generating street-network buffers around schools. Four buffer sizes were established considering ACS threshold distances for children and adolescents from urban and rural areas [30]. The threshold distances are usually calculated using the receiver operating characteristic (ROC) curve analyses [31]. The threshold distances were 1250 m and 1350 m in urban children and adolescents, respectively [30]. The threshold distances of children and adolescents from rural areas were 675 m and 1550 m, respectively [30].

The following measures were used in our analyses: street intersection density (ratio of the number of street intersections to land area of the school neighborhood); number of four or more street intersections (number of this kind of intersection in the land area of the school neighborhood); residential density (ratio of the number of residents to land area of the school neighborhood); and land use mix (diversity of land use types per school neighborhood). The formula used for the land use mix has been used in the IPEN (International Physical Activity and the Environment Network; www.ipenproject.org) methodology. It captures how evenly the square footage of diverse uses is distributed (e.g., residential, retail area, or public services) and ranges from 0 to 1 [33]. Spatially referenced data from different raw data sources were used to create measures. The street network was provided by the Unified Digital Street Map of Andalusia (known in Spanish as CDAU) and by CartoCiudad of the Spanish National Geographic Institute. The spatial distribution of land use and the number of residents were obtained from the ATOM Inspire cadastral service by disaggregation and aggregation operations considering the number of dwellings per building and the census block population data. The software QGIS 3.4 (https://qgis.org/es/site/) was used to generate macro-scale environment variables.

#### Micro-scale variables of the school neighborhood environment

Microscale Audit Pedestrian Streetscapes (MAPS)-Global audit tool [18] was used to measure detailed streetscape features of the school neighborhood. MAPS-Global shows high inter-rater reliability for auditing micro-scale urban form features and pedestrian environments [18]. The items of this instrument are classified in routes, segments (the street between two intersections), and crossings. Considering previous research [34] indicating that sampling 25% of the neighborhood street segments may be sufficient to characterize the pedestrian environment, the block where the school was located was audited. A route was designed surrounding the school on the sidewalk closest to the school building. Data were collected using Google Earth and Google Street View (Google, Inc., Mountain View, CA) imagery, starting at the main entrance of the school. Routes took an average of 97.2 min to be completed (range: 20.0–270.0 min). The final sample included 39 routes (around the school block), 152 street segments, and 247 regulated crossings. On average, audited routes were 609.8 m in length (range: 87.9–1846.1 m).

A member of the research staff with previous experience using MAPS-Global was responsible for training a group of three raters following the certification process indicated by Millstein et al. [35]. Before the data collection, two schools were randomly selected and rated independently. Results were compared to ensure consistency and achieve an inter-rater reliability of 95% agreement or higher. The rest of the 37 schools were randomly assigned to the raters.

The route variables analyzed in the present study were: average number of traffic lanes; number of regulated crossings; average parking street buffer (from “none” =0 to “76–100%” =4); number of traffic calming features (e.g., signs, speed tables, speed humps, etc.); positive streetscape characteristics (subscale composed of 17 items including different measures such as the presence of public transit stops, benches, public trash bins, or bicycle racks); aesthetic and social characteristics (subscale composed of 10 items including different measures related to building maintenance, presence of dog fouling, or the presence of pleasant hardscape features, such as fountains, sculptures, or art); regulated crossing quality (subscale composed of 22 items in relation to, for example, the type of intersection control and signage or the presence of crosswalk amenities, such as crossing aids, marked crosswalk, or refuge islands). As in previous research [25], one of the segment items (i.e., parking street buffer) was adapted to assess both street sides in order to have a more complete evaluation. MAPS-Global single item indicators and subscales scores are described in detail in the work by Cain et al. [18].

#### Socio-demographic characteristics

For the aims of this paper, the following socio-demographic characteristics were considered: participants’ gender and age, and average household income by municipality as a socio-economic status (SES) indicator (Spanish Tax Agency for 2016).

### Data analytic plan

Data processing was performed using Matlab 2019a (Mathworks Inc., Natick, Ma, USA) for Mac OS. Input data were normalized between 0 to 1 (range normalization) to avoid the scale effect in the walkability index computation. A previous study recommended this normalization procedure [36].

Descriptive statistics (e.g., means, standard deviations, and frequencies and percentages for categorical measures) were conducted to examine the distributions of all the measures. In order to know the univariate relationship between the input variables and ACS, linear regression models were computed controlling for age, gender, and SES. The beta value of these models was used to inverse (i.e., 1-value) the input variables that were negatively related to ACS before walking index calculation.

Secondly, we analyzed the associations between built environment factors and ACS to find the best walkability index for the whole sample, and then separately for urban children, rural children, urban adolescents, and rural adolescents. This process was repeated for all the walkability indexes that combined three to six input variables. Walkability indexes in relation to active modes of transport are usually composed of three variables [10, 12, 37]. In the present study, we aimed to analyze indexes with up to six variables. Considering previous research [12, 20], the formula used to compute each walkability index was just the sum of the input variables normalized (range) and inverted (if necessary). Then, each walkability index was related to ACS using a linear model and controlling for age, gender, and SES. In all cases, the one that presented a higher r-value with ACS was selected as the best index. Pearson’s r-value is usually used to select the best estimation model [38].

**Fig. 2:**
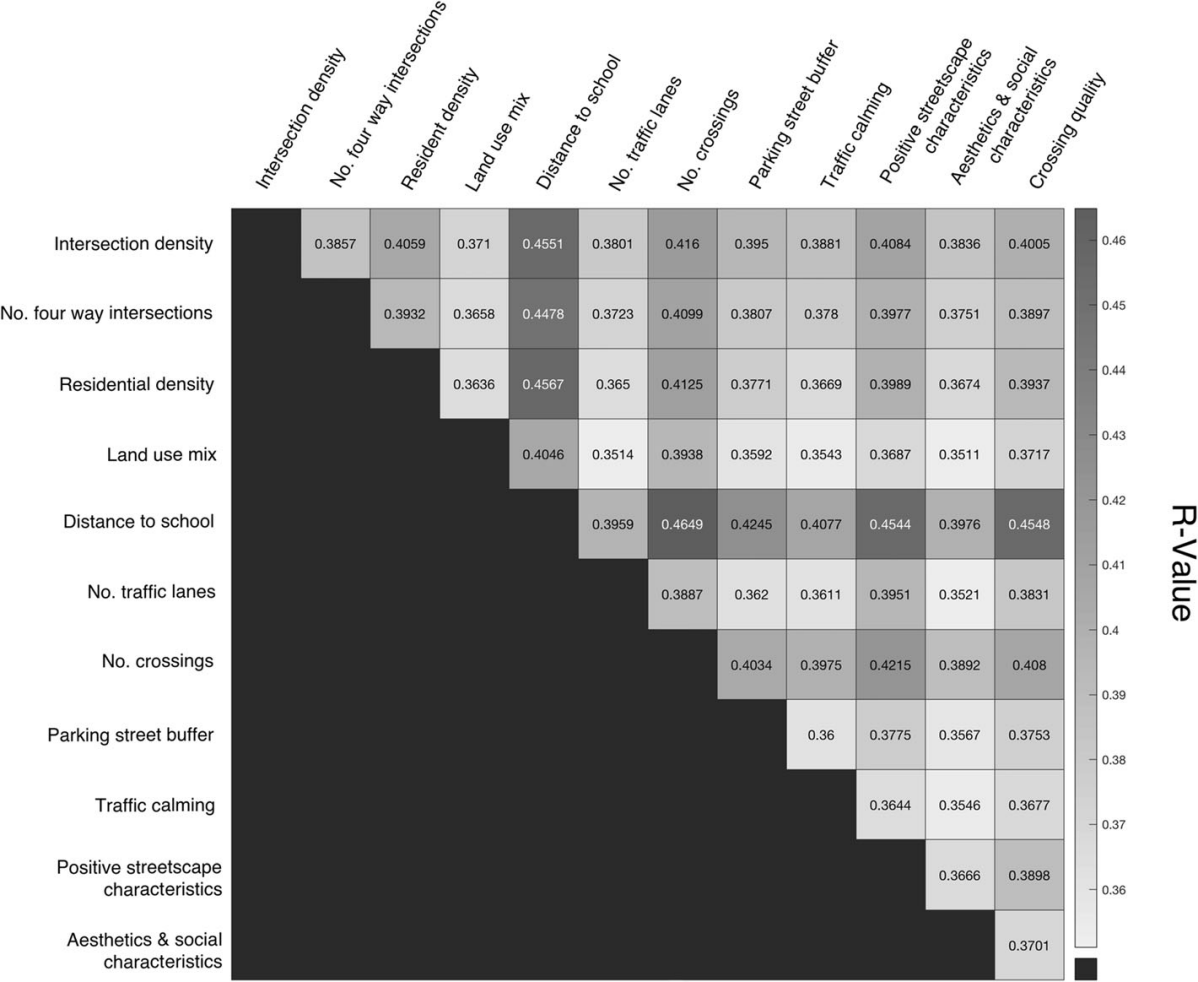
Heatmap of the correlation coefficient between Walkability index and ACS for the whole sample and in function of the variables used to compute it using only two of them. Notes. ACS = active commuting to/from school

There were 66 possibilities to compute walkability index using only two input variables [combinations = nVARIABLES!/(2!*(nVARIABLES-2)!)]. As an example, Fig. 2 reports the r-value of the models obtained to estimate ACS using each walkability index for the whole sample. In this case, the best walkability index using only two input variables was the one based on the sum of the number of regulated crossings and the distance to school.

## Results

**Fig. 3:**
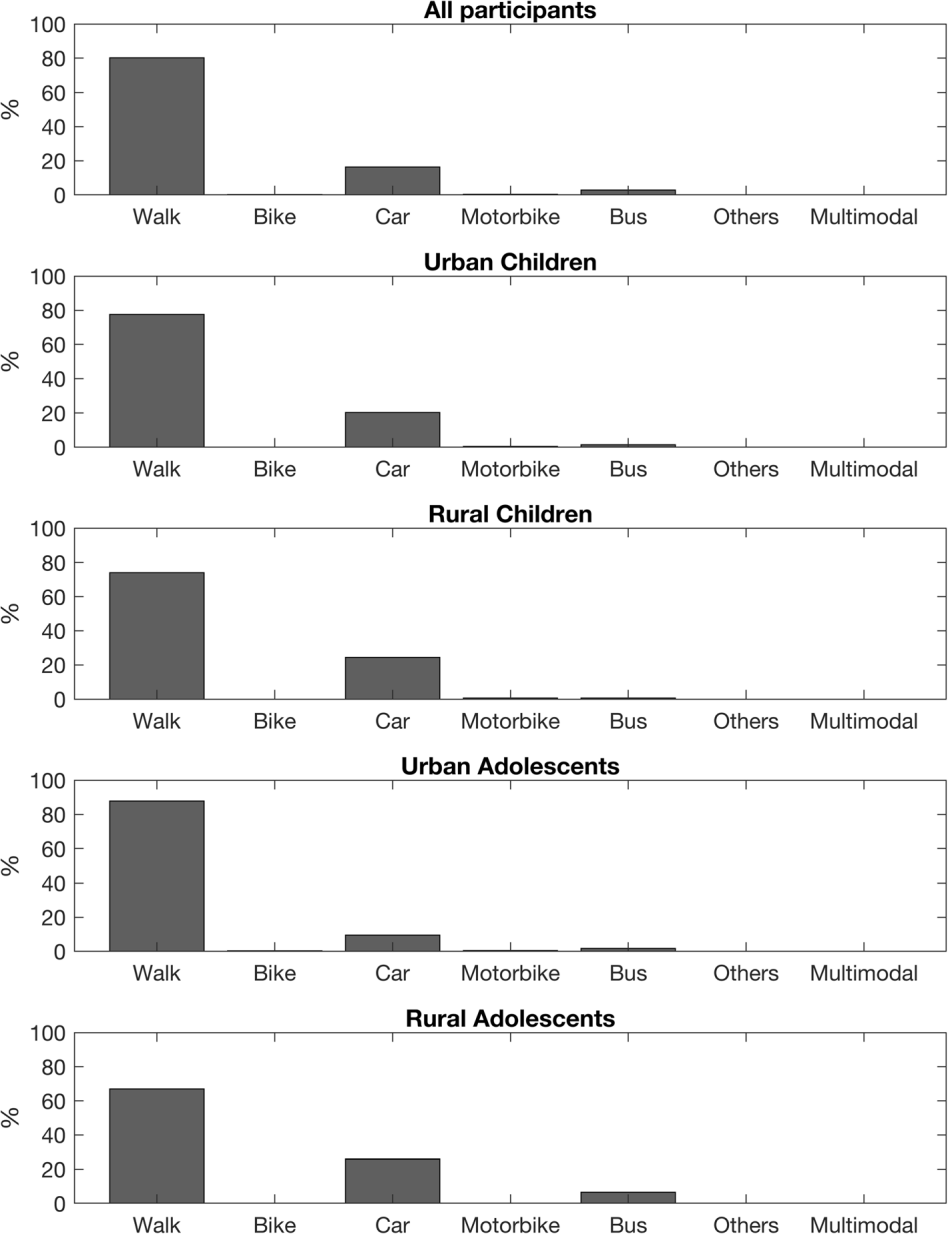
Percentages of trips to and from school by each mode of transport

Descriptive characteristics of the sample by residential area and age are shown in Table 1. Figure 3 gives the percentage of trips to and from school by each mode of transport per week. Most students walked to school (80.12%), whereas the percentage of trips by car was 16.30%. The beta values of the regression models between input variables and ACS are reported in Table 2.

**Table 1:** Descriptive statistics of the sample by residential area and age group

	All	Urban Children	Rural Children	Urban Adolescents	Rural Adolescents
Gender (n (%))					
Male	2330 (50.73)	681(50.0)	108 (51.67)	1042 (50.93)	499 (51.13)
Female	2263 (49.27)	681(50.0)	101 (48.33)	1004 (49.07)	477 (48.87)
Age (years)	12.17 (3.55)	8.20 (2.67)	7.34 (3.66)	14.28 (1.32)	14.31 (1.35)
Distance to school (m)	597.31 (355.64)	504.11 (321.85)	365.36 (210.2)	611.04 (337.87)	748.26 (394.15)
SES (euros/person)	17,862.73 (8912.21)	18,145.9 (2237.15)	14,239.83 (3796.36)	19,785.19 (1227.32)	14,213.33 (4906.29)

*Notes*. SES Socio-economic status

**Table 2:** Beta values (unstandardized regression coefficients) between input variables and ACS by residential area and age group, controlling for gender, age, and SES

Variables	All	Urban Children	Rural Children	Urban Adolescents	Rural Adolescents
***Macro-scale factors***					
Intersection density	2.61**	0.82*	−0.67	1.46**	−3.43*
No. four-way intersections	2.33**	0.28	−0.91	2.22**	−2.69**
Residential density	4.14**	1.31*	−0.84	5.18**	−3.76**
Land use mix	−0.25	−0.38	− 0.50	2.49**	−1.82*
Distance to school	−3.92**	−2.32**	−2.07	− 2.66**	−5.70**
***Micro-scale factors***					
No. traffic lanes	−0.46*	0.12	−0.72	1.55**	−1.34**
No. crossings	2.96**	0.80*	−2.30	2.24**	0.004
Parking street buffer	1.32**	−0.09	−1.26	2.41**	0.63
Traffic calming	0.71**	0.29	−1.12	1.30**	0.39
Positive streetscape characteristics	2.72**	1.18**	−2.14	2.86**	0.31
Aesthetics & social characteristics	0.39*	0.39	0.08	−0.94**	1.37**
Crossing quality	2.26**	0.72	0.26	4.34**	1.18*

*Notes.* *Indicate significant (*p* < 0.05) beta value; **Indicate very significant (*p* < 0.01) beta value

In Table 3, the walkability indexes computed for the entire sample are shown. The performance of the models slightly increases with the number of input variables included in the walkability index. Indeed, the best results were obtained with the index containing four, five, and six variables (i.e., Residential density + Distance to school + No. traffic lanes + Crossing quality + Positive Streetscape characteristics + Intersection density). For urban children, the models that reported better performance were those that included five and six variables (i.e., Residential density + Distance to school + No. crossings + Intersection density + Crossing quality + Positive streetscape characteristics) to compute the walkability index (see Table 4). As Table 4 shows, the walkability index for rural children reported similar goodness of fit values, regardless of the number of variables included in its computation, although the walkability index with four variables showed the best performance (i.e., Intersection density + Positive streetscape characteristics + Distance to school + Parking street buffer).

**Table 3:** Performance of regression models between walkability index and ACS for the whole sample, controlling for gender, age, and SES

No. variables	Walkability index^**a**^	MSE	RMSE	***r***-value
2	[N° crossings + Distance to school]	9.91	3.15	0.46
3	[N° crossings + Distance to school + Positive streetscape characteristics]	9.8	3.13	0.47
4	[Positive streetscape characteristics + Distance to school + No. traffic lanes + Crossing quality]	9.77	3.12	0.48
5	[Residential density + Distance to school +No. traffic lanes + Intersection density + Positive Streetscape characteristics]	9.69	3.11	0.48
6	[Residential density + Distance to school + No. traffic lanes + Crossing quality + Positive Streetscape characteristics + Intersection density]	9.69	3.11	0.48

*Notes*. Normalized values (range normalization) of the variables were used. *MSE* Mean square error, *RMSE* Root mean square error. ^a^The value of the variables Distance to school and No. traffic lanes have been inverted according to the Beta values found in Table 2

**Table 4:** Performance of regression models between walkability index and ACS for urban and rural children, controlling for gender, age, and SES

No. variables	Urban children				Rural children			
	Walkability index^**a**^	MSE	RMSE	***r***-value	Walkability index^**b**^	MSE	RMSE	***r***-value
2	[Positive streetscape characteristics + Distance to school]	8.25	2.87	0.51	[No. traffic lanes + Crossing quality]	8.83	2.97	0.64
3	[Residential density + Distance to school + Intersection density]	8.2	2.86	0.51	[No. traffic lanes + Crossing quality+ Distance to school]	8.76	2.96	0.64
4	[Crossing quality + Distance to school + No. crossings + Positive streetscape characteristics]	8.2	2.86	0.51	[Intersection density + Positive streetscape characteristics + Distance to school + Parking street buffer]	8.72	2.95	0.65
5	[Residential density + Distance to school + Intersection density + Positive streetscape characteristics + Crossing quality]	8.16	2.86	0.52	[No. four-way intersections + crossing quality + Distance to school + No. crossings + Traffic calming]	8.74	2.96	0.64
6	[Residential density + Distance to school + No. crossings + Intersection density + Crossing quality + Positive streetscape characteristics]	8.16	2.86	0.52	[No. four-way intersections + crossing quality + Distance to school + No. crossings + Positive streetscape characteristics + Parking street buffer]	8.77	2.96	0.64

*Notes.* Normalized values (range normalization) of the variables were used. *MSE* Mean square error, *RMSE* Root mean square error. ^a^The value of the variable Distance to school has been inverted according to the Beta values found in Table 2. ^b^The value of the variables No. traffic lanes, Distance to school, Intersection density, Positive streetscape characteristics, Parking street buffer, No. four-way intersections, No. crossings and Traffic calming have been inverted according to the Beta values found in Table 2

For urban adolescents (see Table 5), the walkability index computed with five variables reported the best results (i.e., Residential density + Distance to school + No. four way intersections + Land use mix + Crossing quality). In this case, the improvement in the performance parameters from the model with two variables to the model with five was considerable. Finally, as Table 5 shows, the best models for the rural adolescents were those fitted with the walkability index that included two or three variables (i.e., Distance to school + No. crossings + Intersection density).

**Table 5:** Performance of regression models between walkability index and ACS for urban and rural adolescents, controlling for gender, age, and SES

No. variables	Urban adolescents				Rural adolescents			
	Walkability index^**a**^	MSE	RMSE	***r***-value	Walkability index^**b**^	MSE	RMSE	***r***-value
2	[Crossing quality + Distance to school]	7.28	2.7	0.35	[Intersection density + Distance to school]	14.77	3.84	0.35
3	[Residential density + Distance to school + Crossing quality]	7.21	2.69	0.36	[Distance to school + No. crossings + Intersection density]	14.79	3.84	0.35
4	[Distance to school + No. four-way intersections + Land use mix + Crossing quality]	7.13	2.67	0.38	[Intersection density + Distance to school + No. crossings + Residential density]	15.06	3.88	0.33
5	[Residential density + Distance to school + No. four-way intersections + Land use mix + Crossing quality]	7.07	2.66	0.39	[Intersection density + Distance to school + No. crossings + Positive streetscape characteristics + No. traffic lanes]	15.21	3.9	0.31
6	[Residential density + Distance to school + No. four-way intersections + Land use mix + Positive streetscape characteristics + Crossing quality]	7.08	2.66	0.38	[Residential density + Distance to school + No. traffic lanes + Positive streetscape characteristics + No. four way intersections + Crossing quality]	15.38	3.92	0.30

*Notes.* Normalized values (range normalization) of the variables were used. *MSE* Mean square error, *RMSE* Root mean square error. ^a^The value of the variable Distance to school has been inverted according to the Beta values found in Table 2. ^b^The value of the variables Intersection density, No. four way intersections, Residential density, Land use mix, Distance to school and No. traffic lanes have been inverted according to the Beta values found in Table 2

## Discussion

The main contributions of the current study are that it makes explicit that walkability indexes containing both macro- and micro scale environmental factors are different for urban and rural areas, and even for children and adolescents. Moreover, the walkability components used in the literature seem to correspond better to urban areas than to rural areas.

The built environment characteristics that showed a different role depending on urban or rural areas were: street connectivity indicators (i.e., intersection density and number of four-way intersections) and residential density. These factors were positively related to ACS in urban participants, but negatively in rural participants, which may indicate a higher presence and relevance of these characteristics in urban areas. In addition, it shows that urban areas have higher intersection and residential densities than rural areas. Likewise, it is possible that youths from rural areas avoid walking through highly connected and high density residential neighborhoods because they probably have the busiest streets with the most traffic volume and speed. Highly connected areas have been identified as the ones most used by motorized modes of transport [39]. Moreover, open green areas may be preferred by rural children as a school route instead of residential use areas [40], which are probably busier with more people and heavier traffic. Nevertheless, in a study with US rural adolescents [41], ACS frequency was greater in school neighborhoods with high intersection and residential densities. There seem to be controversial results in rural areas. In our study, street connectivity and residential density measures acted as barriers and not as facilitators of ACS among rural participants. Thus, it is necessary to highlight that diverse criteria are used in rural area definitions across countries (e.g., considering the total population, population density, or some economic indicators) [28, 42, 43], and that the street connectivity and residential density factors seem to be adapted to urban areas. Thus more specific walkability measures should be developed for rural contexts in future studies.

Another result that emphasizes the need to define specific built environment variables for rural areas is that the best walkability indexes are different for urban and rural participants in terms of the number of variables. The number of variables in the best indexes is higher in urban participants compared to rural participants (e.g., 5 variables vs. 2–3 variables in adolescent participants). The lower number of built environment factors included in the best indexes in rural samples would be related, as previously explained, to the need for future research that identifies more specific neighborhood attributes in relation to ACS in rural samples.

In the current study, the walkability indexes were studied for both children and adolescents. The best r-values found in the estimation models for children are higher than those for adolescents (i.e., 0.65 and 0.39, respectively). According to the literature, one possible explanation for this difference may be related to the fact that psychosocial factors might affect the associations between the built environment and ACS among adolescents [19, 44]. In this regard, factors such as social support from peers for ACS seem to be decisive for adolescents when choosing the mode of transport to school [19]. In the case of children, the walkability indexes would better explain ACS because they do not usually have the freedom to choose their mode of transport to school [17]. This decision is typically made by the adults who accompany children to school, and it is more likely to depend on the characteristics of the built environment [17]. Furthermore, the variables included in the walkability indexes with better performance are virtually the same in urban children and adolescents. As expected, and in agreement with previous studies carried out in Europe [10, 17], the majority of the walkability factors included in the best indexes were positively related to ACS (e.g., intersection density, number of four-way intersections, residential density, number of regulated crossings, positive streetscape characteristics, or crossing quality), whereas only distance to school acted as a negative predictor of ACS. In the current study, land use mix only acted as a positive predictor of ACS in urban adolescents, but not in urban children. However, in a previous study carried out with Spanish adolescents [45], none of the land uses examined were significantly related to active commuting in the neighborhood. Future research, probably from a qualitative perspective, should focus on analyzing why a greater variety of destinations on the school route could be positively or negatively related to ACS. In addition, future studies should use an internationally developed set of protocols to measure built environment attributes, such as IPEN protocols, in order to compare studies carried out in different geographical contexts.

Regarding the type of variable included in the walkability index, it is worth noting that all the best walkability indexes include both macro- and micro-scale variables. For instance, in the case of rural children, in addition to intersection density, the best walkability index includes other components that are also negatively related to ACS. One of these components consists of positive streetscape characteristics. Again, it is possible that the areas with more positive characteristics in rural municipalities are busier and have more traffic. Moreover, in our study, an indicator of the presence of cars (i.e., parking street buffer) was also included in the best walkability index as a negative factor for rural children. An implication of our results could be to limit traffic and the presence of cars near rural schools in order to improve ACS, for example, by pedestrianizing areas around schools during children’s entry and exit times. Additionally, the present findings in rural areas would suggest the need to design programs on walking or cycling routes to school, avoiding the busiest streets.

Finally, similar to findings from child-based literature [10, 25], the components of aesthetic and social characteristics of the neighborhood do not seem to be important for children and adolescents to commute actively to school. Aesthetic variables appear to be more related to leisure-time physical activity than to walking/cycling transportation [24]. However, distance to the school is a significant component in all the best walkability indexes. The present results are consistent with the idea that distance to school is considered one of the strongest predictors of ACS, which is why it should be taken into account when analyzing the relationship between the built environment and ACS [19, 46, 47].

One of the implications of our findings for future walkability index designs is the need to create specific measures of macro- and micro- factors related to ACS in rural settings. For this purpose, in-depth interviews or focus groups with experts within the urban design and planning sector could be conducted to identify these measures. Another implication would be the use of statistical protocols to design walkability indexes similar to the one developed in the present study in order to identify the combinations of attributes most strongly related to ACS. Thus, more effective ACS promotion programs could be designed based on the creation of specific indexes for each built environment. In this regard, multicomponent ACS promotion programs should improve the awareness of young people and families about the positive aspects of active transport and create supportive built environments to facilitate ACS. Moreover, school-based ACS programs should consider what the most relevant walkable neighborhood features are, based on the area of residence and age group.

### Study strengths and limitations

The strengths of this study included the use of objective and standardized measurements of macro- and micro-scale environmental factors that allow international comparisons with other countries. To our knowledge, this study is one of the first to examine walkability components related to ACS in both children and adolescents from urban and rural areas. A limitation was that the study was carried out in a region of one country, and so the findings cannot be generalized to other countries. Other limitations include the cross-sectional design of the study, the use of a convenience sample, and employing a school neighborhood evaluation of the micro-scale features of a street-network buffer that only comprises the area surrounding the school.

## Conclusions

Overall, our findings support the conclusion that the relevance of built environment factors for ACS depends on the type of area (i.e., urban or rural) and the age of the sample (i.e., children or adolescents). Our study also demonstrates the importance of including micro-scale environmental factors in walkability indexes. More specific walkability measures for rural areas should be developed in future studies. Interventions focusing on improving built environments to increase ACS behavior need to have a better understanding of the walkability components that are specifically relevant to urban or rural samples.

## Data Availability

The dataset supporting the conclusions of this article is available upon reasonable request to the corresponding author.

## Abbreviations

ACS: Active commuting to and from school
GIS: Geographical information system
IPEN: International Physical Activity and the Environment Network
MAPS: Microscale Audit Pedestrian Streetscapes
MSE: Mean square error
RMSE: Root mean square error
ROC: Receiver operating characteristic curve analyses
SES: Socio-economic status.
